# Pattern-Selection Based Power Analysis and Discrimination of Low- and High-Grade Myelodysplastic Syndromes Study Using SNP Arrays

**DOI:** 10.1371/journal.pone.0005054

**Published:** 2009-04-08

**Authors:** Xiaorong Yang, Xiaobo Zhou, Wan-Ting Huang, Lingyun Wu, Federico A. Monzon, Chung-Che Chang, Stephen T. C. Wong

**Affiliations:** 1 Center for Biotechnology & Informatics and Department of Radiology, The Methodist Hospital Research Institute, Weill Medical College, Cornell University, Houston, Texas, United States of America; 2 School of Statistics and Mathematics, Zhejiang Gongshang University, Hangzhou, China; 3 Department of Pathology, The Methodist Hospital, Weill Medical College, Cornell University, Houston, Texas, United States of America; University of Hong Kong, Hong Kong

## Abstract

Copy Number Aberration (CNA) in myelodysplastic syndromes (MDS) study using single nucleotide polymorphism (SNP) arrays have been received increasingly attentions in the recent years. In the current study, a new Constraint Moving Average (CMA) algorithm is adopted to determine the regions of CNA regions first. In addition to large regions of CNA, using the proposed CMA algorithm, small regions of CNA can also be detected. Real-time Polymerase Chain Reaction (qPCR) results prove that the CMA algorithm presents an insightful discovery of both large and subtle regions. Based on the results of CMA, two independent applications are studied. The first one is power analysis for sample estimation. An accurate estimation of sample size needed for the desired purpose of an experiment will be important for effort-efficiency and cost-effectiveness. The power analysis is performed to determine the minimum sample size required for ensuring at least 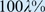 (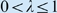) detected regions statistically different from normal references. As expected, power increase with increasing sample size for a fixed significance level. The second application is the distinguishment of high-grade MDS patients from low-grade ones. We propose to calculate the General Variant Level (GVL) score to integrate the general information of each patient at genotype level, and use it as the unified measurement for the classification. Traditional MDS classifications usually refer to cell morphology and The International Prognostic Scoring System (IPSS), which belongs to the classification at the phenotype level. The proposed GVL score integrates the information of CNA region, the number of abnormal chromosomes and the total number of the altered SNPs at the genotype level. Statistical tests indicate that the high and low grade MDS patients can be well separated by GVL score, which appears to correlate better with clinical outcome than the traditional classification approaches using morphology and IPSS sore at the phenotype level.

## Introduction

Myelodysplastic syndromes (MDS) are a heterogeneous group of clonal hematopoietic disorders characterized by peripheral cytopenia, morphologic dysplasia and susceptibility to leukemic transformation [1], [2]. The classification systems include French-American-British (FAB), World Health Organization (WHO) and Internation Prognostic Scoring System (IPSS). Cytogenetic abnormality is one of the most determinants in the prognosis. While a large database of cytogenetic data based on metaphase karyotyping is generated in MDS, and only about 50% clonal abnormalities of primary MDS are detected by conventional cytogenetic studies [3]–[5]. Additionally, there is evidence suggesting that MDS may start with multiple minor clones [6], which may be missed with conventional cytogenetic studies at the initial presentation. The detection of copy number variants and related studies of MDS using single nucleotide polymorphism (SNP) array data has received increasing attention in recent years and is used as a powerful tool for molecular karyotyping.

This article is concerned with our latest MDS study using 250 K Affymetrix SNP arrays. In contrast to other research groups, who used unsorted bone marrow samples [3]–[9], we employ flow cytometry sorting to sort 12 MDS marrow samples into four different fractions: blastic, erythorid, immature myeloid and lymphoid. We also exact oral mucosa DNA from buccal swab as the constitutive DNA samples for each patient. The 250 K SNP microarray analysis is only conducted with fractions, containing enough DNA. Using cell sorting, 35 arrays can be generated from the various fractions derived from 12 MDS patients. This set is split in a test set and normal references consisting 21 and 14 arrays, respectively (See Table S1 in supplementary material for details).

One goal of SNP array studies is to detect the regions of Copy Number Aberration (CNA) in the whole genome. Traditional methods to infer the copy number from a SNP array can be referred to segmentation, modeling and regression approaches. Olshen *et al.* [10] proposed a new algorithm called Circular Binary Segmentation (CBS), which models the data explicitly as a series of segments, with unknown boundaries and heights, and then one can set up some performances or optimize an objective function. In [11], the authors fitted the data to specific models, such as hidden Markov models, which is implemented in the software *CNAG* (Copy Number Analyser for GeneChip®). And in [12] the authors considered LASSO type regression. The principle of SNP arrays is very similar to DNA microarrays. SNP arrays contain hundreds of thousands of immobilized sequences with individual SNPs and only parts of them have CNA. However, CNA of individual SNP or very few consecutive SNPs might be caused by noise. One key question is how patterns with altered SNPs can be selected first. Therefore, we propose to use a so-called Constraint Moving Average (CMA) algorithm. To detect the abnormal regions, the results of this approach are validated by real-time Polymerase Chain Reaction (qPCR). This pattern-selection based method picks up the subsets of copy number altered SNPs from hundreds of thousands of individual SNPs in each array and is afterwards compared with others, computational and intuitional (a more detailed description can be found in Materials and Methods & Discussion and Conclusion). The comparison of the results indicates that our pattern-selection based CMA algorithm has the capability to detect both large and subtle results. In order to see the performance of the CMA algorithm, we also compare the number of abnormal chromosomes (i.e. the chromosomes that contain CNA regions) detected by both CMA and *CNAG* (see the evaluation of the results in Discussion and Conclusion part). In a way, it proves that our CMA algorithm sheds lights on clinical prognosis at the genotype level.

Two independent applications of our CMA algorithm are studied. The first one is the power analysis. An important aspect of experimental design is to determine the number of the samples required in order for the results to be statistically interpretable. It usually refers to power analysis. To perform power analysis, we establish a hypothesis first, and then statistical testing is implemented to decide whether the null hypothesis is accepted or rejected. The power of a test is the probability of getting a statistically significant result, given that the null hypothesis is false (the flowchart is given in Result part). Power is proportional to the sample size, significance level and the effect size, and is also inversely proportional to the variance in the population. Statistical and biological significance can be linked through the use of power analysis. And once given the significance level, the effect size and the desired power, the sample size can be directly estimated for target power.

To estimate the number of the required samples for the purpose of genotype array studies, there already exist some standard methods of power analysis. Like in gene microarray studies, people usually identify the differentially expressed genes across disease subtypes by employing some algorithms, such as Principle Component Analysis (PCA), Significance Analysis of Microarrays (SAM) [13], which are used to solve the typical curse-of-dimensionality problem, or just simply using the *p*-value [14] for the comparison of each gene across the arrays. Then based on the assumption of homogeneous sampling from the entire population of each class, statistical hypothesis test are performed to determine the minimum sample size by using different test statistics. For instance, two group *t*-test based on differences of group means [15], Wilk's lambda score [16], or nonparametric Wilcoxon rank sum test based on differences of rank sums in groups [15]. These algorithms and statistical measurements have already been adopted and proved effectively. However, these methods are only valid in the studies with diseases that have significant homogeneity.

However, the methods mentioned above are due to heterogeneity of the disease invalid in MDS studies. In our experiments, copy number variants in the same regions can hardly be found in SNP arrays from different patients or even in the different hematopoietic fractions (erythroid, myeloid or blastic fraction sorted by flow cytometry) of the same patient. To the best of our knowledge, there is no existing work that attempts to quantify the statistical power in for MDS studies. The major obstacle of such kind of work is that the heterogeneity makes it difficult to design statistical tests and to give an accurate estimated sample size. This motivated us to consider other approaches to deal with this issue.

Based on the CNA regions selected by the CMA algorithm, power analysis can be performed to determine which sample size can ensure that the detected regions are statistically different from the normal references (details are shown in the Results part). Since the heterogeneity of MDS leads to fewer *common CNA regions* (i.e. CNA regions that are in the same location for different samples) among the sample arrays, the required sample sizes may vary in order to make sure that the detected specific regions will be significantly different from the reference in the sense of statistical reliability. Therefore, we formulate the problem to detect at least 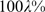 (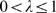) CNA regions at a desired power. The minimum number of array samples required in the experiments can then be estimated by using statistical tests to ensure the statistically significance and expected power. The adjustable proportional parameter 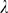 allows us to determine the needed sample sizes for detecting the desired regions. As expected, power increases with increasing sample size with a fixed significance level.

The second application of our pattern-selection based CMA algorithm is to identify the different MDS grades of patients. As we know, the well separated stages of MDS patients (high and low grades MDS patients) can guide the prognosis and survival analysis. The existing methods for discrimination of the grade of MDS patients can refer to both cell morphology and International Prognostic Scoring System (IPSS) score, which belong to methods at the phenotype level. As already mentioned, due to the heterogeneity reflected in the SNP arrays to study this complex group of diseases and consequently the lack of common CNA regions, the traditional classification approaches generally used for analysis at genotype level are no longer available Therefore, we need a new approach to overcome this obstacle. Based on the CMA algorithm, the Risk Likelihood Function and General Variant Level (GVL) score are proposed for each array. The GVL score integrates the information of CNA, such as the number of abnormal chromosomes, the total number of altered SNPs, and return a unified measurement to make the different arrays comparable. Afterwards related analyses according to GVL are considered to discriminate between high and low grade MDS patients. (It is worth to mention that we pay attention to individual patient instead of single arrays here. If we have more than one array for one patient, we need to calculate the GVL for all arrays of this patient, and the average GVL score will be the final GVL score for this patient.) Two group *t*-tests indicate that the GVLs are significantly different for high/low grade (defined by both cell morphology and IPSS score) MDS patients. It gives us a hint that we can set a critical value of GVL as a classification criterion for SNP array analysis. .The classification results achieved with GVL scores at the genotype level appear to be consistent with that of the cell morphology and the IPSS scores. Since the discrimination of the high/low grade MDS is an important issue in the prognosis and the analysis of the chances of survival for the patient, our proposed GVL score gives an analytical criterion for the analysis using SNP arrays.

Our novel contributions are: (i) we develop a new pattern-selection based method to detect the regions of CNA for a heterogeneity disease such as MDS by using sorted bone marrow SNP arrays. Real time PCR results prove that besides large CNA region, the CMA algorithm also presents an insightful discovery of subtle regions; (ii) based on the results of the CMA algorithm, two independent applications are studied. (a) Sample size estimation of the experiment based on selected patterns can be easily done by using statistical. (b) According to the results of the CMA algorithm, the high and low grade MDS patients can be well separated by using the proposed GVL score, which gives a unified measurement to make it comparable among the different arrays. (iii) For comparative analysis, we demonstrate that the number of the abnormal chromosomes detected by CMA is significantly different between patients suffering from high grade and those affected by low grade MDS. Such difference cannot be observed using *CNAG*. The comparison of different algorithms indicates that our method is less complicated and also computable for SNP arrays with high resolution.

## Materials and Methods

### The data-set

Altogether, 35 SNP arrays are generated from 12 MDS patients, 21 and 14 of are treated as test samples and references, respectively. Genomic DNA from each fraction was extracted with Qiagen Allprep RNA/DNA Mini Kit (Qiagen Valencia, CA) and stored at −80°C. Constitutional/control DNA consisted of buccal mucosa and lymphoid fractions of the patients and one marrow sample without evidence of MDS sorted into blastic, erythroid, and myeloid fractions (see Table S1. in supplementary material). The quality and quantity of genomic DNA were assessed by NanoDrop ND-1000 spectrophotometer (NANoDrop Technologies, Wilmington, DE). Genotyping is performed using 250 K NspI SNP-microarray chips (Affymetrix, UK) and processed according to the manufacture's instruction. 250 *ng* of genomic DNA was digested with *Nsp*I for 2 hours at 37°C followed by adaptor ligation, PCR amplification, fragmentation, labeling and hybridization. Three micro-liter of the PCR and 4.5 *ul* of the fragmentation product were electrophoresed to confirm the processing of the DNA. The Affymetrix 450 fluidics station and the Affymetrix gene scanner were used to wash, stain and scan the arrays. Signal intensity and SNP calls are analyzed using *CNAG* and *Genotyping Console*.

Due to the high variability of the mean intensities across different SNP arrays, normalization is necessary to make different SNP arrays comparable [17]. In this study, the sums of intensities of the perfect match probes for alleles A and B are normalized using invariant set method by [18], [19]. After normalization, the log-2-ratio features are extracted using the ‘best-fit’ method also used in *CNAG* [20].

### Pattern-selection based CMA for CNA region detection

Consider the pair of tumor and normal samples from the same patient (but different tissue), the SNP is genotype call conflicting in these pair samples, if the genotype of one SNP is homozygous in the normal sample, but heterozygous in the tumor sample, or both are homozygous but have different alleles. In order to reduce the risk of false positives or false negatives in the final results, SNPs with conflicting genotype call between samples and references are filtered right at the beginning. Table 1 displays the number of SNPs with conflicting genotype calls considering all arrays. It also the probabilities of such conflicts occur due to random errors, which are calculated assuming that the observed conflicting SNPs completely caused by random errors follow the binomial distribution *B*(*n*, *p*), with the parameters *n* denoting the total number of SNP arrays (in our case, *n* = 35) and *p* being calculated as follows
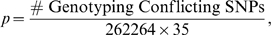
(1)where 262264 is the total number of the SNPs in one array. Then the probability of the observed SNPs in all arrays caused by random error can then be calculated by
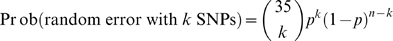
(2)The smaller the probability is, the higher is the possibility that the genotype call is wrong.

**Table 1:** Results for genotype conflicting analysis for 35 arrays.

**# conflicting arrays**	**0**	**1**	**2**	**3**	**4**	**5**
**# SNPs**	171057	38094	26262	10919	7069	3770
**Prob. of random errors**		0.362959	0.142516	0.036324	0.006756	0.000977
**# conflicting arrays**	**6**	**7**	**8**	**9**	**10**	**>10**
**# SNPs**	2384	1242	734	386	216	181
**Prob. of random errors**	0.000114	1.12E-05	9.22E-07	6.56E-08	4.07E-09	2.33E-10

E.g. there are 3770 genotyping conflicting SNPs, each of which appears in 5 arrays, and if such conflicts are just due to random error (i.e. can not be regarded as wrong genotyping calls), the probability is 0.000977.

By virtue of the small probabilities that indicate the conflicting SNPs appearing at least in 3 of the arrays, only the remaing 235413 (171057+38094+26262) SNPs will be analyzed in the next stage.

In the next step, we use *Genotyping Console* to automatically obtain the list of SNPs spanning over the regions of known CNA or segmental polymorphism. These SNPs were reported in the literatures, indicating the occurrence of CNA in healthy population (population without MDS). By filtering those SNPs, 170795 SNPs in each array are finally left for the further analysis.

On account of CNA in part of the arrays, we need to detect those regions first. Among the methods for copying number estimation, *CNAG* and *dChip* receive more concerns, especially *CNAG*, because *CNAG* uses a different normalization method which can remove the baselines of raw data. Both of them use the Hidden Markov Model (HMM). However in the HMM, only the current observation and the previous hidden state are employed to infer the current hidden state, which makes it easily susceptible to false detections due to strong noisy data. Here we propose a straightforward method making use of the fact that the copy number is a constant in one region. Copy number variations of single or fewer SNPs may not be correct because noise in the data and therefore we focus on detecting a pattern of copy number variations in this study. In order to use the contextual cues, a so-called Constraint Moving Average (CMA) algorithm with length five is performed. For all the maintained SNPs in genome, ranging from chromosome 1 to chromosome 22, every five consecutive SNPs are taken into account as one region. We check the average log-2 ratio of each region in samples, by comparing them with their corresponding references. We select a specific region as an abnormal one if it satisfies the following condition ***C***.

***C*** (1) The mean of the log-2 ratios for a set of consecutive five SNPs in a region across the chromosomes satisfies either >0.35 or <−0.35, which correspond to the critical values of a copy number of three and one, respectively.
(2) The standard deviation (*SD*) of those five log-2 ratios should be <0.15.

**Figure 1:**
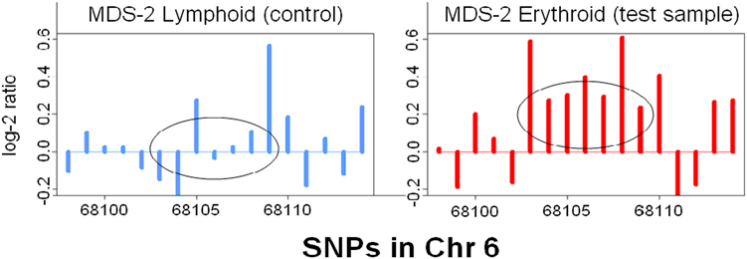
Illustration of the CMA algorithm. MDS-2 Lymphoid is the reference, and MDS-2 Erythroid is the test sample. In the test sample, the average log-2 intensities of every five consecutive SNPs in the circled region are higher than 0.35, with the small SDs (< = 0.15). Selected overlapping regions are merged into a large region.

The threshold of log-2 ratio here is ±0.35, the same as in [17]. Finally, all the selected CNA regions in the samples are recombined in order to exclude the overlapping cases. Figure 1 illustrates the CMA algorithm for MDS-2 Myeloid.

The CMA algorithm reduces the complexity of the model, and fewer parameters are employed, which makes it robust and easy to be performed and computable for high resolution SNP arrays. In addition, the mean of the log-2 ratio gives an intuitional hint of the real copy number variations and together with the restriction on the standard deviation. It avoids false positives caused by strong noises. Compared with the results of *CNAG*, the proposed CMA algorithm presents an insightful discovery of subtle regions, which could be “missed” by other approaches (like *CNAG* and CBS). The results are supported by real time PCR. Table 2 shows the region located in chromosome 7q34 in MDS-3, starting from the 85974^th^ SNP and ending at 85978^th^ SNP. Blast and Erythroid fractions are used as test samples, and Lymphoid is the corresponding reference. These three fractions are from the same patient. The region covers the gene *FOXP2*. CMA results indicate that, compared with the reference Lymphoid, only Erythroid has deletion in this region. The result is supported by real-time PCR (see Table 2; see also in [21]). However, this CNA region is missed by *CNAG* and CBS.

**Table 2:** Output of the CMA algorithm results for a region located in chromosome 7q34 and the corresponding real-time PCR results.

Sample	Fraction	Mean	SD	PCR
MDS-3	Lymphoid (control)	0.0951	0.1220	1
	Blast (test sample)	0.1869	0.1724	0.91
	Erythroid (test sample)	0.3563	0.1229	0.65

Three fractions from the same patient are displayed. The Lymphoid is the normal reference. Blast and Erythroid serve as test samples. The log 2 ratio behaviors them are different. As normal one, the log 2 ratio of Lymphoid is closed to 0. There is a significant loss in Erythroid, but for Blast, the log 2 ratio is not low enough. The real time PCR of Lymphoid is normalized as 1. Comparing with the reference, Erythroid is concluded as copy number aberration. However, such abnormality can not be observed in Blast.

## Results

Our CMA algorithm has the capability to detect both large and subtle regions. The comparison of the results with other algorithms and the choice of parameters will be discussed in the Discussion and Conclusion section. The CMA approach has two independent applications. The first one is the power analysis to estimate the required sample size that ensures statistical difference between the detected regions and the normal references. We also want to pay attention to the MDS patients. Based on the results of the CMA algorithm, we propose to distinguish the high grade MDS patient from the low grade one by using the GVL scores. These two applications are exhibited in the following two subsections.

### Minimum sample size estimation by power analysis

**Figure 2:**
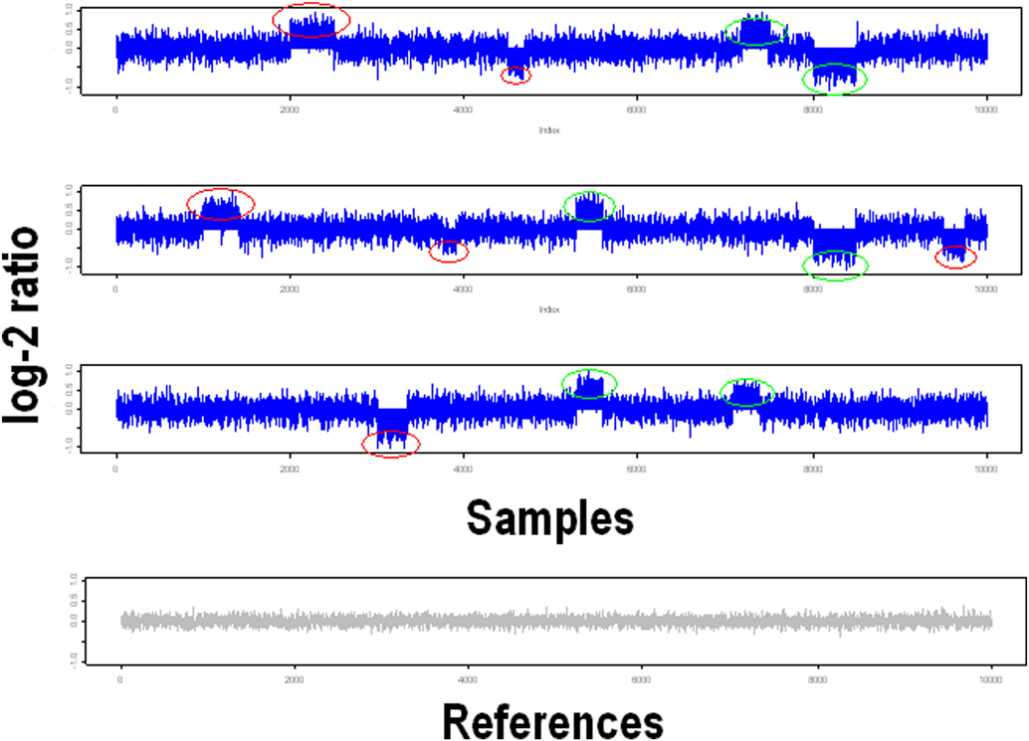
CNA regions selected by CMA algorithm. Compared with the references, the regions with circles indicate the CNA regions. For different samples, the CNA regions may occur in the different locations. Some appear repeatedly (the ones with green circles), and some others rarely occur (the ones with red one).

Using the pattern-selection based CMA algorithm, the CNA regions can be detected for each array. Figure 2 is a sketch map of the CNA regions detected by CMA algorithm. For each selected CNA regions all across the genome, the behavior may not be accordant in different arrays. Some appear repeatedly for different sample arrays (CNA regions marked with green circle), whereas some others emerge rarely (CNA regions marked with red circle). The goal of power analysis is to determine the minimum sample sizes required to ensure that the detected CNA regions are statistically different from normal references, (rather than occurring randomly). For a specifically selected CNA region, we use the average log-2 intensity to estimate the copy numbers. Then we compare the average intensity of the same regions between samples and references. The hypothesis of the power analysis can be formulated as the testing of the null hypothesis 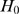 against the alternative hypothesis 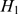.

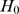
: For the specific region, the average intensity between samples and references is same (*log-2* ratio = 0),

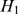
: For the specific region, the average intensity between samples and references is significantly different (*log-2* ratio≠0).

To estimate the sample size, usually one refers to power analysis. There are four quantities in the power analysis, sample size, effect size, significance level 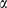 and power. With three known, the fourth will be determined. In statistics, the terms *Type I error* (also referred as significance level 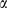, of false positive) and *Type II error* (also known as 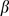 error, of false negative) are used to describe possible errors made in a statistical process (the target power is usually defined as 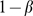). They are usually called as “two sources of error”, namely,

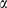
: The error of rejecting a hypothesis that should have been accepted;

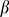
: The error of accepting a hypothesis that should have been rejected.

Another principal challenge posed in the field of power analysis is how to define the effect size. The effect size is a measure of biological significance. It gives the difference between the results predicted by the null hypothesis and the actual state of the population being tested. In a clinical study, when the interest is to target the power for different effect sizes, sample size can be estimated to ensure that the endpoint with the smallest effect size is sufficiently powered with a fixed significance level 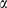. Thus, if the measurements are meaningful on a practical level, it is encouraged to give the effect size by one's experience. One of most accepted opinions to determine the effect size is the one mentioned in [22], where 0.2 indicates of a small effect, 0.5 a medium and 0.8 a large effect size.

Due to the limited amounts of SNP arrays used in our MDS study, it is difficult to define an appropriate effect size empirically. Therefore, we prefer to use the standardized effect size from [23], defined as
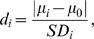
(3)for the selected abnormal region 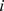, where 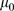 is the critical value set as 0 (the log-2 ratio is 0 if the average intensity is not different between the samples and the references) in our case, 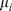 and 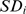 are the mean and standard deviation of all sample for a specific region, respectively.

**Figure 3:**
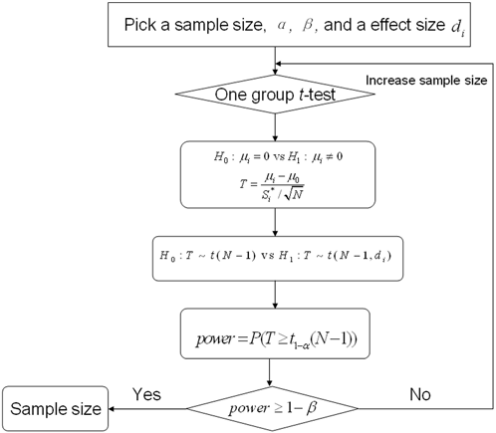
Power analysis algorithm for the estimation of the minimum sample size.

When 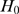 is true, the test statistic 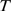 (see the definition in Figure 3.) follows *t* distribution with degree of freedom 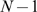 (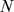 is the number of total test arrays); when 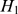 is true, 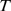 follows the non-central *t* distribution with degree of freedom 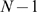 and non-central parameter 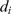. Given a type I error 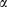 and a type II error 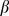, and the formulation of effect size 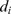, the power analysis algorithm for the estimation of minimum sample size follows the flowchart showing in Figure 3, where 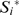 is the estimation standard deviation of test samples.

Since the repeatability number of each detected CNA region is different, the effect size varies. It results in altered sample sizes for the detection of the different specific regions. For some regions, the effect sizes are so small that we can hardly see any CNA region in the test samples. Sometimes, only a fraction of the CNA regions are receiving attentions according to the purposes of experiments. Especially for those frequently appearing regions, only a fewer samples will be required for statistical interpretation. While for rarely emerging regions, we need a huge sample size to ensure the statistical significance of tests. Therefore, the sample size depends on the desirability of the study. It does not necessarily require identification of all abnormal regions at the same time. Accurate sample size estimation will be important to an efficient and economical study design. To implement it, we first collect the abnormal regions derived by CMA algorithm. For each detected region 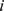, we calculate the corresponding effect size 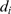, and sort them decreasingly. A proportion parameter 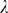 (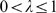) now is employed, and the number of sample arrays required to detect at least 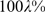 can be obtained following the methods shown in the flowchart of Figure 3, with the effect size chosen as the *k*-th one in the decreasing sequence, where *k* denotes the smallest integer larger than 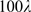. Usually, for frequently appearing regions, the effect sizes are much larger than that of others, which implies that smaller sample sizes are needed.

**Figure 4:**
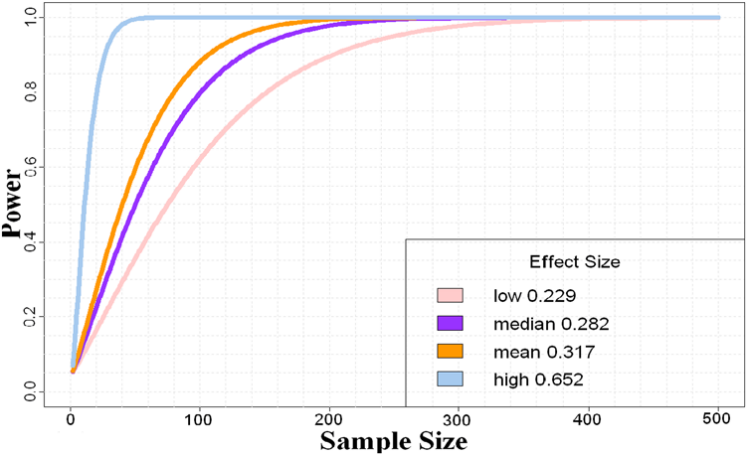
Power curves and sample size estimation under different views of effect sizes. The significance level is set as 0.05.

According to our CMA algorithm, in total 1117 of the detected regions are non-overlapping. Power analysis is executed according to the flowchart in Figure 3. We calculate the effect sizes (See equation (3)) for each region. The sorted effect sizes range from 0.0004 to 1.062, the median and mean are 0.282 and0.317, respectively. 889 out of 1117 are less than 0.5, which implies that most of them are small ones. The average of those less than 0.5 (the 889 ones), is only 0.229 (low), while the average of the rest is 0.652 (high). Figure 4 shows the sample size under different views of effect size with fixed significance level at 0.05.

Table 3 summarizes the sample sizes needed for a detection proportion from 40% to 80%. With the estimated sample sizes, target power can be reached under the most frequently used significance level 0.1, 0.05, 0.01. As expected, an increase of the desired proportion increases requires more and more samples. Since for most of the detected regions, the corresponding effect sizes calculated by equation (3) are small ones, we suggest that one can choose effect sizes from 0.2 to 0.3 can be chosen for a rough estimation.

**Table 3:** Sample size estimation to detect at least 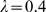, 0.5, 0.6, 0.7 and 0.8 truly altered regions for the desired power up to 0.8 and 0.9, with different significance level.

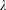	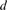	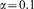		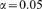		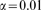	
		P = 0.8	P = 0.9	P = 0.8	P = 0.9	P = 0.8	P = 0.9
0.4	0.344	54	74	69	91	102	129
0.5	0.283	79	109	101	134	150	190
0.6	0.230	118	163	150	200	224	284
0.7	0.171	215	296	272	364	406	516
0.8	0.108	528	731	671	897	998	1271

(P: Power).

### The discrimination of patients with high and low grade MDS

The discrimination of high and low grade of MDS patient is an important issue in the prognosis and survival analysis of MDS studies. Biologists use cell morphology and the IPSS score to determine the assessment of patient's MDS severity. Those kinds of classification are important to clinical survival analysis in the future. However, at the genotype level, to the best of our knowledge, there is no relative research focusing on this issue. As an application of the proposed CMA algorithm, we first define the Risk Likelihood Function and the General Level (GVL) score as in (4) and (5); then the GVL score will be used for the discrimination between the high and low grade MDS. Some statistical tests show that, high and low grade MDS can be well separated by the definition of GVL. The difference between the two groups is significant, which implies that it can give a quantitative criterion for the classification when using SNP arrays.

#### The Risk Likelihood Function

The Risk Likelihood Function defined as follows, takes account of two aspects, one is the chromosome abnormalities, and other one is the number of altered SNPs.
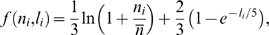
(4)where 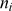 is the number of abnormal chromosomes in array *i*, 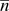 stands for the average abnormal chromosome of the test samples, and 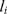 denotes the total number of the abnormal SNPs across the genome in array *i*, setting the denominator as 5 in the power due to the length of the CMA algorithm (also see Materials and Methods). The first term focuses on the proportion of abnormal chromosomes across the whole array. Since the slowly varying function 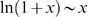, as 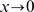, when there are a fewer the abnormal chromosomes (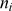 is small), it will not change much; but for the larger one, it varies slowly. The slowly varying function will eliminate the influence of extremely large or small 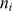. The second part is an analogy to the Haldane map function defined in hidden Markov model [18]. Besides the altered SNPs, the risk function also includes the number of abnormal chromosomes of individual SNP array, and the overall average number of abnormal chromosomes of the all population. Obviously, the array with more abnormal chromosomes and more altered SNPs will suffer high risk.

#### The General Variant Level (GVL)

The General Variant Level presenting the log-2 ratio variant with the Risk Likelihood Function can be defined as
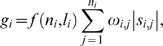
(5)where 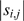 describes the absolute value of average intensities of the *j*-th chromosome in array *i*. The GVL integrates the risk function and quantity of CNA regions with weights 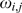. It depicts the overall information of the SNP array. Here we take the absolute values of the average intensities, because our hypothesis focuses on whether the copy number changes or not for the samples changes in contrast to the references. We only need to see how far they deviate from normal. We give the weights 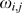 to the absolute value of average intensities according to the proportion of the number of the abnormal regions appearing in all samples. For instance, in the Myeloid fraction of MDS-7, there are three abnormal chromosomes, chromosome 3, 4 and 18. In the total of 21 samples, 4 arrays contain abnormal regions in chromosome 3, 6 arrays in chromosome 4, and 5 arrays in chromosome 18. Hence, in this case the weight 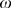 is 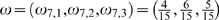.

Though there are only a few common regions among the sample arrays, using the proposed CMA algorithm and the GVL score, we make the arrays comparable. Thereby, high and low grade MDS patients can be well separated. Table 4 displays the discrimination of 12 severe and less affected patients by morphology and IPSS score. The 12 patients are separated into two groups by morphology; one is high grade (H), and other is low grade (L). By IPSS, there are four different grades, i.e. low (L), inter-median I (Int-1), inter-median II (Int-2), and high (H). We merge the low and inter-median I as a low group; inter-median II and high as a high group in IPSS classification. Then for each array, we calculate its GVL.

**Table 4:** The average GVL of MDS patients and the discrimination between the high grade MDS and the low grade MDS by both cell morphology and IPSS score.

Sample	Fraction	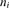	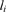	GVL	Average	High/Low by morphology	IPSS
MDS-1	Blast	8	365	0.3871	0.3835	H	Int-1
	Erythroid	6	53	0.3799			
MDS-2	Myeloid	12	74	0.3581	0.4011	H	Int-2
	Erythroid	15	174	0.4441			
MDS-6	Blast	4	16	0.3336	0.2725	H	Int-2
	Erythroid	3	15	0.3119			
	Myeloid	1	6	0.1719			
MDS-8	Blast	5	1610	0.3635	0.3561	H	Int-1
	Erythroid	3	875	0.3286			
MDS-10	Myeloid	6	4586	0.3742	0.3742	H	H
MDS-3	Blast	0	0	0	0.1123	L	Int-1
	Myeloid	0	0	0			
	Erythroid	4	30	0.3369			
MDS-4	Myeloid	0	0	0	0.0895	L	L
	Erythroid	1	5	0.1790			
MDS-5	Erythroid	1	5	0.1808	0.1808	L	L
MDS-9	Erythroid	1	5	0.1939	0.1939	L	L
MDS-11	Myeloid	0	0	0	0	L	Int-1
MDS-12	Myeloid	0	0	0	0	L	Int-1
MDS-7	Blast	15	198	0.4264	0.3842	**uncertain**	Int-1
	Myeloid	3	38	0.3421			

A GVL of zero implies that there is no selected abnormal region in the corresponding arrays.

From Table 4, we notice that the number of abnormal chromosomes for the low grade MDS cases is usually less than 5, in contrast to the high grade ones. Also, the total number of the altered SNPs is much smaller for low grade MDS (< = 30) than that of high grade one. The argument of the classification results by cell morphology is acceptable in the sense that to set critical value of the average GVL score of a patient as 0.2. MDS-7 is a special case. The morphological classification shows no significant evidence to which grade it belongs. It is claimed as uncertain. However, by our CMA algorithm and the GVL score calculation, its two fractions exhibit different behaviors, with lower GVL score in the Myeloid and higher GVL score in Blast. However, the average GVL score seems high. Another interesting case is MDS-1. Although it classified to high grade by cell morphology, according to IPSS score, it is a low grade MDS. However, we can see a large scale of deletion in chromosome 7 for both Blast and Erythroid fractions; hence the average GVL for this patient is high. Since the GVL of MDS-1 is in favor of the classification result by morphology, we have reason to define MDS-1 as high grade.

Based on the classification results by cell morphology and IPSS score, we perform the two-group *t*-tests to compare the GVL between high and low grade MDS. Results are listed in Table 5. The tests include two cases; one is without the uncertain MDS-7, and the other is with MDS-7. The *p*-value shows that the GVL scores of two groups are significantly different, which indicates that high and low grade MDS can be well separated by using the proposed GVL score. Even though the GVL results tend to have a minor discrepancy compared with IPSS, the *t*-test results can still prove the significance difference between two groups. Therefore, the results of GVL appear to correlate better with clinical outcome than the traditional classification approaches using cell morphology and IPSS score.

**Table 5:** Two group *t*-test results for the discrimination of MDS grade of using General Variant Level score in the sense of cell morphology and IPSS.).

	Morphology			IPSS		
Without MDS-7	*t*-value	df	*p*-value	*t*-value	df	*p*-value
	9.3989	9	0.0001	4.2432	9	0.0022
With MDS-7	*t*-value	df	*p*-value	*t*-value	df	*p*-value
	5.6028	10	0.0002	3.6182	10	0.0047

The cutoff value of copy number one and three in *CNAG* is −0.35 and 0.35, and the window size of moving average is 5. The *t*-value is the value of statistics in the *t* test, and df is the degree of freedom of the test.

## Discussion

With the proposed CMA algorithm, we detect the CNA regions using the mean and SD of every five consecutive SNPs as criteria. Actually, computing the mean in a region can be regarded as a constant regression to predict the real log-2 ratios. We have also tried different methods than constant regression, such as local linear regression, quadratic regression to select the CNA regions by the threshold of mean and SD. Most of the selected CNA regions of CMA algorithm can be included by performing the local linear regression, because the local linear regression will not change the mean of a region. However, due to the correction of SD, it almost abolishes the restriction of SD, which leads to overestimation, especially in the case of heavy noise data. Since the quadratic regression will essentially change both the mean and SD of a specific regions and cannot give an intuitionist view of the log-2 ratio, hence it is not robust enough.

Statistical approaches for analyzing copy number data are aimed at detecting the regions of genomic alteration. One alternative method is to model the data explicitly as a series of segments, with unknown boundaries and heights, and then one can set up some performances or optimize an objective function, like proposed in [19] to use a new algorithm called Circular Binary Segmentation (CBS). Others have fitted the data as specific models, such as hidden Markov model in [18], and LASSO type regression in [20]. In essence, our algorithm is a kind of regression-based method. We compare it with other methods to evaluate its efficiency and the accuracy the results. First of all, we use the LASSO based penalized least square estimation discussed in [20]. We define the same cutoff values as the authors. However, the algorithm fails when applied to our data. It takes more than 6 hours for each sample array to run, but nothing can be found. The examples shown in [20] are CGH data with about 2000 points in each array, whereas our data set contains 250 K single SNPs in each array. The failure might occur because the LASSO based method is time-consuming for high resolution SNP array. Table 4 compares the selected abnormal regions in chromosome 7 by CMA (regression based method) algorithm with *CNAG* (HMM based method) and CBS (segmentation based method). When the abnormal regions are large, the results are consistent for the different methods. However, although HMM used in *CNAG* gives a reasonable inference of copy numbers, it does not treat the log-2 ratio in a local region as a pattern, some individual SNPs are reported as abnormal ones, which are not reliable. Sometimes CBS can find many small regions with very fewer altered SNPs, but the SDs of these regions may be much larger than what we expected, which results in a high number of false positives. Furthermore we want to take a look at the mean and the SD of the remaining regions that were found by *CNAG* and CBS, but missed with the CMA algorithm in Table 6. As displayed in the brackets, the CMA algorithm drops those regions mainly because that some of them consist just single altered SNPs, which might be caused by the noise in the data; and also because some dissatisfy the thresholds (the fluctuations (SD) in a local region are too large). This implies that the proposed CMA algorithm is robust. Another advantage is that the CMA algorithm dramatically reduces the complexity of the model, and enables an insightful discovery for subtle discovery.

**Table 6:** Comparison of selected abnormal regions in chromosome 7 by CMA algorithm, *CNAG* and CBS. Y and N denote if the region is selected or not, respectively.

Arrays	Regions	CMA	*CNAG*	CBS
MDS-8 B	Chr 7 monosomy **(validated by PCR)**	Y	Y	Y
MDS-8 E	Chr 7 monosomy **(validated by PCR)**	Y	Y	Y
MDS-1 B	7q34–7q36.1	Y	Y	Y
MDS-3 E	7q34 **(validated by PCR)**	Y	N	N
MDS-1 B	7p21.3	Y	N	N
MDS-2 E	7p14.2	Y	N	N
MDS-1 E	7q14.1 (mean = 0.56; SD = 0.47)	N	Y	N
MDS-1 E	7q34 (mean = 0.25; SD = 0.20)	N	Y	N
MDS-2 E	7p31.1 (mean = 0.24; SD = 0.34)	N	Y	N
MDS-2 M	7p31.3 (single SNP)	N	Y	N
MDS-2 M	7q34 (mean = 0.29; SD = 0.28)	N	Y	N

B: Blast; E: Erythroid.

Next we want to compare the CNA regions discovered with the CMA algorithm and *CNAG*. The average number of chromosomes containing CNA regions and the corresponding standard deviations are listed in Table 7. The parameters in *CNAG* are set consistent with CMA, i.e. the length of moving average is 5 (the default is 10); the cutoff values of copy number one and three are −0.35 and 0.35, respectively (the default are −0.49 and 0.30, respectively). Using the CMA algorithm, the classification results for cell morphology show that the number of abnormal chromosomes that CNAs (for cases with multiple fractions tested, the average abnormal chromosomes of all fractions are used) is significantly smaller (*p*-values are close to zero) in low grade MDS cases than it is in patients with high MDS cases using CMA. Such difference cannot be observed by *CNAG*. The same conclusions can be made in IPSS scores classification. In addition, more comparisons with different parameters in *CNAG* are made. Notice that a chromosome may only have one single altered SNP according to the *CNAG*'s output; therefore, we also exclude those chromosomes in the comparison in order to reduce the false positives. The conclusion is quite similar as what we display is Table 7 (See Table S2, S3, S4 in supplementary material for details).

**Table 7:** Copy number aberrations comparison of CMA algorithm and *CNAG* (MDS-7 is excluded).

CMA	H		L		*t*-value	df	*p*-value
	mean	SD	mean	SD			
Morphology	5.93	2.83	0.64	0.56	7.07	9	0.0001
IPSS	6.22	3.67	1.85	2.44	4.72		0.0011

*CNAG*	H		L		*t*-value	df	*p*-value
	mean	SD	mean	SD			
Morphology	14.30	2.96	9.17	5.03	2.01	9	0.0753
IPSS	12.38	1.67	11.18	5.68	0.35		0.7344

Two-group *t*-test are performed under the null hypothesis, that mean of two groups are no significant different.

At last we want to discuss the length of CMA algorithm, as it is a critical parameter for the success of our study. Notice that the overlapping regions selected by the CMA algorithm will be merged to large and non-overlapping regions; therefore, the length of final copy number aberration regions may not be fixed at five. In this study, we can regard the length five as an initial length. Our choice is based on the real-time PCR results, indicating that the copy number aberration region will not be selected, if we change the length to six, due to the dissatisfaction of both mean and SD for the log-2 ratios of six consecutive SNPs'. The mean and the SD for a length of five are −0.356269 and 0.1229, respectively. However, they change to −0.296754 and 0.1826 for a length of six. Furthermore, we think that if the initial length is too short, we may find much more false positive regions. By trying different lengths, we conclude that the proposed one of five is the most suitable and it can be the minimum length for the selection of CNA regions. However, the user may change the initial length appropriate to the data. If the user has prior knowledge about the data, we recommend that the initial length should be chosen according to the prior information.

## Supporting Information

Table S1 Details of the used SNP arrays. The references are marked in shade.
(0.05 MB DOC)

Table S2 Copy number aberrations comparison of CMA algorithm and CNAG (MDS-7 is excluded). The cutoff value of copy number one and three in CNAG is −0.35 and 0.35, and the window size of moving average is 5 (chromosomes with only single altered SNP excluded). Two-group t-test are performed under the null hypothesis that the means of two groups are no significant different.
(0.03 MB DOC)

Table S3 Copy number aberrations comparison of CMA algorithm and CNAG (MDS-7 is excluded). The cutoff value of copy number one and three in CNAG is −0.49 and 0.30 (default setting), and the window size of moving average is 5. Two-group t-test are performed under the null hypothesis that the means of two groups are no significant different.
(0.03 MB DOC)

Table S4 Copy number aberrations comparison of CMA algorithm and CNAG (MDS-7 is excluded). The cutoff value of copy number one and three in CNAG is −0.49 and 0.30 (default setting), and the window size of moving average is 5 (chromosomes with only single altered SNP excluded). Two-group t-test are performed under the null hypothesis that the means of two groups are no significant different.
(0.03 MB DOC)

## References

[1] Delforge M (2003). Understanding the pathogenesis of myelodysplastic syndromes.. Hematol J.

[2] Hofmann WK, Lubbert M, Hoelzer D, Phillip Koeffler H (2004). Myelodysplastic syndromes.. Hematol J.

[3] Haase D, Fonatsch C, Freund M, Wormann B, Bodenstein H (1995). Cytogenetic findings in 179 patients with myelodysplastic syndromes.. Ann Hematol.

[4] Haase DGU, Schanz J, Pfeilstocker M, Nosslinger T, Hildebrandt B (2007). New insights into the prognostic impact of the karyotype in MDS and correlation with subtypes: evidence from a core dataset of 2124 patients.. Blood.

[5] Sole F, Luno E, Sanzo C, Espinet B, Sanz GF (2005). Identification of novel cytogenetic markers with prognostic significance in a series of 968 patients with primary myelodysplastic syndromes.. Haematologica.

[6] Gondek LP, Haddad AS, O'Keefe CL, Tiu R, Wlodarski MW (2007). Detection of cryptic chromosomal lesions including acquired segmental uniparental disomy in advanced and low-risk myelodysplastic syndromes.. Exp Hematol.

[7] Greenberg P, Cox C, LeBeau MM, Fenaux P, Morel P (1997). International scoring system for evaluating prognosis in myelodysplastic syndromes.. Blood.

[8] Mohamedali A, Gaken J, Twine NA, Ingram W, Westwood N (2007). Prevalence and prognostic significance of allelic imbalance by single nucleotide polymorphism analysis in low risk myelodysplastic syndromes.. Blood.

[9] Gumus G, Sunguroglu A, Tukun A, Sayin DB, Bokesoy I (2002). Common fragile sites associated with the breakpoints of chromosomal aberrations in hematologic neoplasms.. Cancer Genet Cytogenet.

[10] Olshen AB, Venkatraman ES, Lucito R, Wigler M (2004). Circular binary segmentation for the analysis of array-based DNA copy number data.. Bioinformatics.

[11] Fridlyand J, Stransky AM, Pinkel D, Albertson DG, Jain AN (2004). Hidden Markow models approach to the analysis of CGH data.. J Multivar Anal.

[12] Huang T, Wu B, Lizardi P, Zhao H (2005). Detection of DNA copy number alterations using penalized least squares regression.. Bioinformatics.

[13] Tusher VG, Tibshirani R, Chu G (2001). Significance analysis of microarrays applied to the ionizing radiation response.. Proc of the Natl Acad Sci U S A.

[14] Golub TR, Slonim DK, Tamayo P, Huard C, Gaasenbeek M (1999). Molecular classification of cancer: Class discovery and class prediction by gene expression monitoring.. Science.

[15] Thomas JG, Olson JM, Tapscott ST, Zhao LP (2001). An efficient and robust statistical modeling approach to discover differentially expressed genes using genomic expression profiles.. Genome Res.

[16] Johnson RA, Wichern DW (1992). Applied Multivariate Statistical Analysis.

[17] Zhao X, Li C, Paez JG, Chin K, Janne PA (2004). An integrated view of copy number and allelic alterations in the cancer genome using single nucleotide polymorphism arrays.. Cancer Res.

[18] Li C, Wong WH (2001). Model-based analysis of oligonucleotide arrays: model validation, design issues and standard error application.. Genome Biol.

[19] Li C, Wong WH (2001). Model-based analysis of oligonucleotide arrays: expression index computation and outlier detection.. Proc Natl Acad Sci U S A.

[20] Nannya Y, Sanada M, Nakazaki K, Hosoya N, Wang L (2005). A robust algorithm for copy number detection using high-density oligonucleotide single nucleotide polymorphism genotyping arrays.. Cancer Res.

[21] Huang WT, Yang X, Zhou X, Monzon FA, Wen J (2009). Multiple distinct clones may co-exist in different lineage in myelodysplastic syndromes.. Leukemia Research.

[22] Cohen J (1992). A power primer.. Psychological Bulletin.

[23] Cohen J (1988). Statistical power analysis for the behavioral sciences (2nd Ed.).

