# Supplementary material for: Pattern-Selection Based Power Analysis and Discrimination of Low- and High-Grade Myelodysplastic Syndromes Study Using SNP Arrays
Source: PLoS One. 2009 Apr 8;4(4):e5054. doi: 10.1371/journal.pone.0005054 (PMC2662412; doi:10.1371/journal.pone.0005054)
Supplement: Table S1 — Details of the used SNP arrays. The references are marked in shade. (0.05 MB DOC) [file pone.0005054.s001.doc]

**Table S1.** Details of the used SNP arrays. The references are marked in shade.

| **Patient** | **SNP array** | **Fraction** | **Test sample or Reference** |
| --- | --- | --- | --- |
| **MDS1** | Array1 | Blast | Test sample |
|  | Array2 | Erythroid | Test sample |
|  | Array3 | Lymphoid | Reference |
| **MDS2** | Array4 | Myeloid | Test sample |
|  | Array5 | Erythroid | Test sample |
|  | Array6 | Lymphoid | Reference |
| **MDS3** | Array7 | Blast | Test sample |
|  | Array8 | Myeloid | Test sample |
|  | Array9 | Erythroid | Test sample |
|  | Array10 | Buccal | Reference |
|  | Array11 | Lymphoid | Reference |
| **MDS4** | Array12 | Myeloid | Test sample |
|  | Array13 | Erythroid | Test sample |
|  | Array14 | Lymphoid | Reference |
| **MDS5** | Array15 | Erythroid | Test sample |
|  | Array16 | Buccal | Reference |
| **MDS6** | Array17 | Blast | Test sample |
|  | Array18 | Myeloid | Test sample |
|  | Array19 | Erythroid | Test sample |
|  | Array20 | Lymphoid | Reference |
|  | Array21 | Buccal | Reference |
| **MDS7** | Array22 | Blast | Test sample |
|  | Array23 | Erythroid | Test sample |
|  | Array24 | Lymphoid | Reference |
| **MDS8** | Array25 | Blast | Test sample |
|  | Array26 | Erythroid | Test sample |
|  | Array27 | Lymphoid | Reference |
| **MDS9** | Array28 | Erythroid | Test sample |
|  | Array29 | Buccal | Reference |
| **MDS10** | Array30 | Myeloid | Test sample |
|  | Array31 | Lymphoid | Reference |
| **MDS11** | Array32 | Myeloid | Test sample |
|  | Array33 | Buccal | Reference |
| **MDS12** | Array34 | Myeloid | Test sample |
|  | Array35 | Lymphoid | Reference |
