# Supplementary material for: Pattern-Selection Based Power Analysis and Discrimination of Low- and High-Grade Myelodysplastic Syndromes Study Using SNP Arrays
Source: PLoS One. 2009 Apr 8;4(4):e5054. doi: 10.1371/journal.pone.0005054 (PMC2662412; doi:10.1371/journal.pone.0005054)
Supplement: Table S2 — Copy number aberrations comparison of CMA algorithm and CNAG (MDS-7 is excluded). The cutoff value of copy number one and three in CNAG is −0.35 and 0.35, and the window size of moving average is 5 (chromosomes with only single altered SNP excluded). Two-group t-test are performed under the null hypothesis that the means of two groups are no significant different. (0.03 MB DOC) [file pone.0005054.s002.doc]

**Table S2.** Copy number aberrations comparison of CMA algorithm and *CNAG* (MDS-7 is excluded). The cutoff value of copy number one and three in CNAG is -0.35 and 0.35, and the window size of moving average is 5 (**chromosomes with only single altered SNP excluded**). Two-group t-test are performed under the null hypothesis that the means of two groups are no significant different.

| MA | H | | L | | *t-*value | df | *p*-value |
| --- | --- | --- | --- | --- | --- | --- | --- |
|  | mean | SD | mean | SD |  |  |  |
| Morphology | 5.93 | 2.83 | 0.64 | 0.56 | 7.07 | 9 | 0.0001 |
| IPSS | 6.22 | 3.67 | 1.85 | 2.44 | 4.72 |  | 0.0011 |
| *CNAG* | H | | L | | *t*-value | df | *p*-value |
|  | mean | SD | mean | SD |  |  |  |
| Morphology | 7.73 | 2.55 | 4.63 | 2.53 | 2.00 | 9 | 0.0766 |
| IPSS | 6.88 | 1.17 | 5.72 | 3.35 | 0.57 |  | 0.5826 |
