# Supplementary material for: Pattern-Selection Based Power Analysis and Discrimination of Low- and High-Grade Myelodysplastic Syndromes Study Using SNP Arrays
Source: PLoS One. 2009 Apr 8;4(4):e5054. doi: 10.1371/journal.pone.0005054 (PMC2662412; doi:10.1371/journal.pone.0005054)
Supplement: Table S3 — Copy number aberrations comparison of CMA algorithm and CNAG (MDS-7 is excluded). The cutoff value of copy number one and three in CNAG is −0.49 and 0.30 (default setting), and the window size of moving average is 5. Two-group t-test are performed under the null hypothesis that the means of two groups are no significant different. (0.03 MB DOC) [file pone.0005054.s003.doc]

**Table S3.** Copy number aberrations comparison of CMA algorithm and *CNAG* (MDS-7 is excluded). The cutoff value of copy number one and three in CNAG is -0.49 and 0.30 (default setting), and the window size of moving average is 5. Two-group t-test are performed under the null hypothesis that the means of two groups are no significant different.

| MA | H | | L | | *t-*value | df | *p*-value |
| --- | --- | --- | --- | --- | --- | --- | --- |
|  | mean | SD | mean | SD |  |  |  |
| Morphology | 5.93 | 2.83 | 0.64 | 0.56 | 7.07 | 9 | 0.0001 |
| IPSS | 6.22 | 3.67 | 1.85 | 2.44 | 4.72 |  | 0.0011 |
| *CNAG* | H | | L | | *t*-value | df | *p*-value |
|  | mean | SD | mean | SD |  |  |  |
| Morphology | 14.37 | 1.53 | 10.19 | 5.24 | 1.30 | 9 | 0.2259 |
| IPSS | 12.78 | 1.34 | 11.21 | 4.68 | 0.54 |  | 0.6023 |
